# Threshold-Aware Link-Graph Compression with Per-Sensor Observed-Packet Coverage Constraints and Temporal Robustness in Urban LoRaWAN Networks

**DOI:** 10.3390/s26206562

**Published:** 2026-10-16

**Authors:** Viacheslav Osadchyi, Andrii Bondarchuk, Olha Bondarenko, Mykola Pyroh, Hlib Kryvolapov, Yaroslav Kryvolapov

**Affiliations:** 1 Faculty of Economics and Management, Borys Grinchenko Kyiv Metropolitan University, 04053 Kyiv, Ukraine; v.osadchyi@kubg.edu.ua; 2 Faculty of Information Technologies and Mathematics, Borys Grinchenko Kyiv Metropolitan University, 04212 Kyiv, Ukraine; 3 Faculty of Information Technology, Taras Shevchenko National University of Kyiv, 60 Volodymyrska Street, 01033 Kyiv, Ukraine; olha.bondarenko@knu.ua (O.B.); m.pyroh@knu.ua (M.P.); y.kryvolapov@knu.ua (Y.K.)

**Keywords:** LoRaWAN, LPWAN, link-graph compression, observed-packet coverage, fairness-aware selection, dynamic programming, temporal robustness, urban sensing, Internet of Things

## Abstract

**Highlights:**

**What are the main findings?**

- On the studied deployment, a threshold-independent fairness-aware link ordering matched the exact minimum-cardinality optimum on all 1000 tested requirement pairs, whereas aggregate-coverage-only selection matched it on 4%. However, under heterogeneous observed-packet volumes, the reusable fairness-aware ordering exhibited mean cardinality gaps of 25.4 and 48.8 links for 50 and 100 synthetic sensors, respectively, whereas the threshold-dependent multi-cover greedy method attained the exact minimum in all tested cases.
- A single subset that remains feasible in every observed month requires 19 of 30 links, compared with mean month-specific optima of 9.24–11.59 links.

**What are the implications of the main findings?**

- Explicit per-sensor observed-packet coverage constraints prevent high aggregate coverage from masking insufficient coverage of individual sensors and should be considered when every active sensor must remain represented in the monitoring layer.
- Link budgets quoted from a single aggregated observation window understate the cost of a deployable fixed subset, and newly activated sensors require an explicit discovery rule.

**Abstract:**

In multi-gateway LoRaWAN deployments, a single uplink packet may be received through several sensor–gateway links, creating link-graph redundancy. We formulate compression as minimum-cardinality selection under aggregate and per-sensor observed-packet coverage constraints. The sensor-separable structure enables an exact dynamic program to certify optima and benchmark a reusable fairness-aware greedy ordering, a threshold-dependent multi-cover greedy, and a coverage-only ablation. Evaluation uses 1,232,813 uplinks observed by at least 1 gateway over 17 calendar months. On this deployment, proposed and multi-cover methods matched the minimum across 1000 threshold pairs. Coverage-only was optimal for 4%, with mean and maximum gaps of 5.84 and 8 links. These methods returned different equal-cardinality subsets for 506 pairs. The proposed subset improved worst-month coverage slack in 437 cases and met both requirements in more months in 53 cases, never in fewer. Retrospective monthly optimization required 9.24–11.59 links on average, versus 19 for an all-month-feasible subset. In a 14-month prospective evaluation, the expanding-window policy achieved pass rates of 78.6%, 57.1%, and 50.0% with three or four reconfigurations of at most two links each, and an unseen sensor caused a common cold-start failure. These results quantify trade-offs among link budget, future-month compliance, and subset stability.

## 1. Introduction

The Long-Range Wide-Area Network (LoRaWAN) is a widely used low-power wide-area network (LPWAN) technology for Internet of Things (IoT) applications [1], including urban and campus deployments [2,3]. In multi-gateway configurations, a single uplink packet may be received simultaneously by several gateways, after which the duplicates are forwarded to the network server and removed during the deduplication phase. This architecture improves reception reliability, but it also introduces redundancy in the sensor–gateway link graph.

Aggregate observed-packet coverage weights unique observed packets rather than sensors. Consequently, a sensor that contributes a smaller share of the observed traffic has less influence on the aggregate metric, and a selected link subset may preserve a high fraction of all packets while providing poor or zero coverage for that sensor. Previous LoRaWAN studies have reported unequal packet-reception performance among end devices and have examined differentiated packet-delivery requirements across device groups [4,5]. We therefore include an explicit lower bound on the observed-packet coverage of every active sensor alongside the aggregate observed-packet coverage requirement. The aggregate metric alone would not enforce this per-sensor condition.

This raises the practical challenge of reducing the number of sensor–gateway relationships considered by downstream monitoring and decision-support procedures without a meaningful loss of observed-packet coverage. Since the selection in this study is performed only after packet reception, the deployed gateways and their standard LoRaWAN forwarding behavior remain unchanged. The smaller selected set reduces the number of sensor–gateway relationships that must later be retained, compared and updated during monitoring, diagnostic or rule-based processing, without affecting radio airtime or the duplicate traffic already forwarded by the gateways.

Long-term variability in urban LoRaWAN reception additionally raises the question of whether a subset selected on past data continues to satisfy the constraints in future months. A period-aggregated optimum can hide month-to-month changes in gateway visibility, and newly activated sensors introduce cold-start conditions that a past-only selector cannot resolve. This work therefore considers both the compression of the whole-period link graph and the temporal behavior of the selected subsets, including retrospective month-specific reoptimization and prospective policies that are fitted on past months and evaluated on the next month.

In this work, the problem of compressing the LoRaWAN link graph is formulated as minimizing the number of selected links while simultaneously satisfying constraints on aggregate observed-packet coverage and on the minimum observed-packet coverage among all sensors. The main contributions of this paper are as follows:

1. a minimum-cardinality formulation of LoRaWAN link-graph compression with simultaneous aggregate and per-sensor observed-packet coverage requirements, together with two related temporal formulations: a month-specific optimum and a robust all-month static optimum;
2. an exact dynamic program that exploits the sensor-separable reception structure and provides certified whole-period and month-specific reference optima;
3. a comparison of a compact threshold-independent fairness-aware ordering with a threshold-dependent capped-utility multi-cover greedy, a coverage-only greedy, and static link-ranking baselines;
4. an empirical evaluation over 1000 threshold pairs, including gaps from the exact minimum cardinality and the multiplicity of optimal subsets, extended by a temporal-quality comparison of the equal-cardinality subsets produced by the proposed ordering and the multi-cover greedy;
5. a temporal evaluation over 17 calendar months that covers retrospective month-specific reoptimization, robust all-month static selection, rolling prospective policies with a same-month oracle reference, an analysis of the new-sensor cold start caused by the appearance of a previously unseen sensor, and the reconfiguration stability of the compared policies, complemented by a synthetic scalability evaluation over larger sensor and gateway configurations.

The remainder of this paper is organized as follows. Section 2 reviews related measurement studies, gateway-placement work, adaptive configuration methods, redundant multi-gateway forwarding and submodular fair-cover formulations. Section 3 describes the dataset, the link-graph model, the three optimization formulations, the compared selection methods and the temporal evaluation protocol. Section 4 reports the whole-period, temporal and synthetic scalability results. Section 5 discusses the scope, limitations and operational implications of the findings, and Section 6 concludes.

## 2. Related Work

### 2.1. LoRaWAN Measurement Studies

Several public datasets with LoRaWAN measurements in urban and campus deployments have been reported, including the LoRaWAN at the Edge (LoED) dataset [6], an urban LoRaWAN fingerprint-localization dataset from Antwerp [2], the Oulu campus dataset [3], urban LoRaWAN measurements [7], and a large-scale study of signal propagation losses in urban LoRa networks [8]. These studies demonstrate radio-channel variability in real deployments. The present work uses the University of Virginia (UVA) LoRaWAN dataset [9] because it provides packet-level reception metadata for individual gateways over a multi-month observation window, which is required to analyze both the redundancy in the sensor–gateway link graph and its variability between calendar months.

### 2.2. Gateway Placement and Related Optimization Problems

LoRaWAN gateway placement has been studied using graph-based methods [10] and hybrid metaheuristics [11]. Separately, multi-gateway throughput has been optimized by tuning random-access backoff parameters [12]. These studies address network planning or radio-access performance rather than post-reception link-graph compression. This paper instead assumes that the network is already deployed and selects a minimal subset of existing sensor–gateway links that preserves aggregate and worst-sensor observed-packet coverage, and then it examines how this selection has to be adapted or re-fitted across months.

### 2.3. Adaptive LoRaWAN Configuration Under Changing Link Conditions

Adaptive-data-rate studies adjust the spreading factor and the transmit power in response to changing channel conditions and mobility, while multi-gateway approaches additionally exploit macro-diversity across gateways [13,14]. These methods optimize radio parameters, convergence, reliability or airtime. The present work keeps the radio configuration fixed and instead studies the temporal transferability of a minimum-cardinality sensor–gateway link subset under aggregate and per-sensor observed-packet coverage constraints.

### 2.4. Redundant Multi-Gateway Forwarding

Redundant forwarding in multi-gateway LoRaWAN has also been addressed directly. Fardin et al. [15] proposed a device–gateway coordination protocol that assigns each IoT device to a single gateway, avoiding repeated delivery of the same frame to the network server and allowing part of the processing to be moved towards the edge. Al-Awami [16] considered duplicate gateway traffic using interflow network coding with the aim of reducing the amount of redundant data carried from gateways to the network server.

The present study begins after these forwarding operations have already taken place, leaving gateway assignment and standard LoRaWAN forwarding unchanged while using the collected reception records to select which sensor–gateway relationships are kept in the graph for downstream monitoring and decision-support procedures.

### 2.5. Multi-Submodular and Fair Cover

Nguyen and Thai introduced the Minimum Robust Multi-Submodular Cover for Fairness problem, in which a minimum-cardinality set must satisfy multiple monotone submodular thresholds even after element removals [17]. Its non-robust case with zero removals is closely related to our threshold-constrained formulation. Fair Submodular Cover instead requires a balanced composition of the selected set while satisfying a common submodular coverage threshold [18]. Utility–fairness balancing [19] and streaming submodular maximisation with knapsack constraints [20] address related but distinct settings.

### 2.6. Positioning of This Work

Existing LoRaWAN optimization studies operate at several parts of the network. Gateway-placement methods select physical deployment sites to improve network coverage and performance  [10,11]. Multi-gateway throughput optimization can instead tune random-access backoff parameters [12], while adaptive-configuration methods modify spreading factors, transmission power or other radio parameters in response to channel conditions  [13,14]. Multi-gateway redundancy has also been addressed through device–gateway coordination [15] and through reduction of duplicate gateway-to-server traffic [16].

The present work starts from the packet-reception history of an already deployed network and selects a minimum-cardinality subset of the observed sensor–gateway relationships used in later monitoring and decision-support processing. The simultaneous aggregate and per-sensor observed-packet coverage constraints connect this selection problem with the fair-cover literature [17,18,19,20].

The selected subset must satisfy both an aggregate coverage threshold and a separate coverage threshold for every active sensor. The sensor-separable reception structure is exploited to obtain an exact dynamic-programming reference, which is used to evaluate both reusable and threshold-dependent greedy strategies. The temporal analysis further distinguishes retrospective month-specific reoptimization, a robust all-month static solution and prospective policies fitted exclusively on past months.

To the best of our knowledge, no previous LoRaWAN study has jointly considered minimum-cardinality selection of observed sensor–gateway reception links, simultaneous aggregate and per-sensor observed-packet coverage constraints, an exact sensor-separable reference solution and prospective evaluation of the selected subsets across multiple calendar months.

## 3. Materials and Methods

### 3.1. UVA LoRaWAN Dataset

This work uses the public, long-term UVA LoRaWAN dataset [9], collected on the University of Virginia campus from 9 February 2024 to 11 June 2025. Timestamps were converted to Coordinated Universal Time (UTC) before observations were assigned to calendar months. The dataset spans 17 calendar months, with the first and the last months being incomplete, corresponding to approximately 16 months of actual observation.

The dataset contains 2,091,512 metadata records for uplink packet receptions from ten sensor nodes by three gateways, which are designated in this paper as A, B and C. For each packet reception, the following information is available: timestamp, received signal strength indicator (RSSI), signal-to-noise ratio (SNR), spreading factor, bandwidth, frequency, airtime and packet counter. Since the same uplink packet can be received by several gateways, a packet is identified by the pair (sensor, counter). In the processed data, no such pair occurred at more than one timestamp or in more than one calendar month. Although this diagnostic does not establish protocol-level uniqueness of the identifier, it found no evidence of counter reuse and reduces the risk of merging distinct transmissions after resets or rollovers. After deduplication based on this identifier, 1,232,813 unique packets received by at least one gateway were obtained.

The set of active sensors is not constant over the observation window. Sensors sensor01–sensor09 are observed from the beginning of the dataset, while sensor10 first appears in May 2025 and remains active in June 2025. In June 2025, sensor03 has no observed packets. In the temporal evaluation, the active-sensor set for each month is therefore defined as the set of sensors with at least one observed uplink packet in that month. The first appearance of sensor10 is treated as a new-sensor cold-start event.

### 3.2. Link-Graph Model

Let S denote the set of sensors (|S|=10), G the set of gateways (|G|=3), and L={(s,g):s∈S,g∈G} the set of candidate sensor–gateway links, with |L|=30. Let P denote the set of observed unique uplink packets. For each packet p∈P, let L(p)⊆L be the subset of links on which this packet was observed.

For each sensor s∈S, let $P_{s}=\left\{p\in P:\mathrm{sensor}\left(p\right)=s\right\}$ denote the observed packets associated with sensor *s*, and let $L_{s}=\left\{\left(s,g\right):g\in G\right\}$ denote the set of links incident to sensor *s*.

For a chosen subset X⊆L, a packet *p* is considered covered if L(p)∩X≠Ø. Let C(X) denote the set of packets covered by *X*, and let $C_{s}\left(X\right)=C\left(X\right)\cap P_{s}$ denote the covered packets of sensor *s*.

Let M denote the set of calendar months in the observation window. For a month m∈M, let $P^{\left(m\right)}$ be the set of packets received in month *m*, let $P_{s}^{\left(m\right)}=P^{\left(m\right)}\cap P_{s}$ be the packets from sensor *s* in month *m*, and let $S^{\left(m\right)}=\left\{s\in S:\right|P_{s}^{\left(m\right)}\left|>0\right\}$ be the sensors active in month *m*. Whole-period quantities correspond to the union over all months.

### 3.3. Problem Formulation

#### 3.3.1. Observed-Packet Coverage Measures

Coverage is calculated from the uplink packets recorded by at least one gateway during the corresponding observation window. Packets missed by all gateways are absent from the dataset, so their number is unknown and they cannot be included in the denominator used below. The resulting measures describe the fraction of unique observed packets preserved by a selected link subset and do not provide an estimate of the end-to-end delivery probability of all transmissions generated by the sensors.

We first define aggregate and sensor-level coverage for a fixed observation window. The aggregate observed-packet coverage is given in Equation (1):

(1) $$P\left(X\right)=\frac{\left|C(X)\right|}{\left|P\right|}\cdot 100\;\%.$$

For each sensor s∈S, its individual coverage is given in Equation (2):

(2) $$S_{s}\left(X\right)=\frac{|C_{s}\left(X\right)|}{|P_{s}|}\cdot 100\;\%.$$

The worst-sensor coverage is then given in Equation (3):

(3) $$S_{\min}\left(X\right)=\min_{s\in S}S_{s}\left(X\right).$$

Because covered-packet counts are integers, the optimization and threshold-stopping routines use the equivalent count targets in Equation (4):

(4) $$N_{P}=\left\lceil \frac{P_{\min}}{100}\left|P\right|\right\rceil ,\;N_{s}=\left\lceil \frac{S_{\min}^{*}}{100}\left|P_{s}\right|\right\rceil .$$

The ceilings are evaluated using exact decimal arithmetic. Thus, $P\left(X\right)\geq P_{\min}$ is tested as $\left|C\left(X\right)\right|\geq N_{P}$, and each sensor-level condition is tested as $|C_{s}\left(X\right)|\geq N_{s}$. The same conversion is applied to the corresponding packet totals of each monthly or past-only fitting window.

#### 3.3.2. Whole-Period Compression Problem

We formulate the link-graph compression problem for the whole-period reception patterns as the problem of finding the minimum number of links that satisfy both thresholds:

(5) $$\begin{array}{cc} \mathrm{minimise}\; & \left|X\right|\\ \mathrm{subject}\;\mathrm{to}\; & P\left(X\right)\geq P_{\min},\\ \; & S_{\min}\left(X\right)\geq S_{\min}^{*},\\ \; & X\subseteq L. \end{array}$$

The coverage function |C(X)| is monotone submodular [21]. If the per-sensor constraint is removed, $P_{\min}=100\;\%$, and the model is restricted to a single sensor with an arbitrary number of incident links, Problem (5) reduces to the classical set-cover problem and is NP-hard [22].

For the considered LoRaWAN structure, however, every packet and every candidate link belong to exactly one sensor. Hence, the individual coverage constraints are sensor-separable and the aggregate coverage is additive across sensors. Equivalently, the formulation is a LoRaWAN-specific non-robust instance of minimum multi-submodular cover [17] with an additional separable structure, which is exploited in Section 3.4.5.

#### 3.3.3. Month-Specific and Robust All-Month Formulations

For the temporal analysis, two related formulations are used. Given a month *m* and thresholds $\left(P_{\min},S_{\min}^{*}\right)$, the month-specific optimum $k^{*}\left(m\right)$ is the value of (5) evaluated with P, $P_{s}$ and S replaced by their month-*m* counterparts $P^{\left(m\right)}$, $P_{s}^{\left(m\right)}$ and $S^{\left(m\right)}$. A distinct link subset can therefore be selected for each month, and the resulting cardinalities describe the lower bound on the link budget attainable with unrestricted month-specific adaptation.

The robust all-month static optimum constrains a single link subset *X* to satisfy both thresholds in every month simultaneously as specified in Equation (6):

(6) $$\begin{array}{cc} \mathrm{minimise}\; & \left|X\right|\\ \mathrm{subject}\;\mathrm{to}\; & P^{\left(m\right)}\left(X\right)\geq P_{\min},\;m\in M,\\ \; & S_{\min}^{\left(m\right)}\left(X\right)\geq S_{\min}^{*},\;m\in M,\\ \; & X\subseteq L, \end{array}$$

where $P^{\left(m\right)}$ and $S_{\min}^{\left(m\right)}$ denote the aggregate and worst-sensor coverage restricted to month *m*. This formulation characterizes the cost of temporal robustness without reconfiguration: only one subset is deployed, but it must remain feasible across all months in the observation window.

### 3.4. Link Selection Methods

#### 3.4.1. Baselines

We first consider one random control and two static ranking baselines. Random-*k* (random selection of *k* links using a fixed seed of 42, averaged over 100 repetitions) serves as a control.

For the Top-*k* reliability baseline, links are ranked by the composite score in Equation (7):

(7) $$r\left(\ell \right)=0.30\;\tilde{C}\left(\ell \right)+0.20\;\tilde{R}\left(\ell \right)+0.20\;\tilde{N}\left(\ell \right)+0.15\;{\tilde{T}}_{R}\left(\ell \right)+0.15\;{\tilde{T}}_{N}\left(\ell \right).$$

The weights were chosen heuristically to reflect three engineering considerations. The largest weight, 0.30, is assigned to the direct packet-reception fraction because packet preservation is the primary objective of the ranking. The mean RSSI and SNR receive equal weights of 0.20 each to represent average link quality without privileging either radio metric, while the temporal-stability terms receive equal weights of 0.15 each to penalize links whose apparently good average quality is not stable across months. The weights sum to one and are used only to define a transparent engineering ranking baseline rather than a trained or theoretically optimized model. Here, $\tilde{C}\left(\ell \right)$ is the min–max normalized fraction of the corresponding sensor’s packets received on link *ℓ*, while $\tilde{R}\left(\ell \right)$ and $\tilde{N}\left(\ell \right)$ are the min–max normalized mean RSSI and SNR. The quantities ${\tilde{T}}_{R}\left(\ell \right)$ and ${\tilde{T}}_{N}\left(\ell \right)$ are defined as one minus the min–max normalized temporal coefficient of variation, computed as the standard deviation of the monthly mean RSSI or SNR values divided by the absolute value of their mean across active months. All components lie in [0,1].

For the Top-*k* RSSI/SNR baseline, links are ranked by $q\left(\ell \right)=0.5\;\tilde{R}\left(\ell \right)+0.5\;\tilde{N}\left(\ell \right)$. Ties in both static rankings are resolved by the lexicographic order of link identifiers.

#### 3.4.2. Fairness-Aware Greedy

At each step, the algorithm adds the link that simultaneously maximizes the increase in total coverage and the increase in the worst-sensor coverage metric. For the current set *X* and a candidate ℓ∉X, we define the evaluation

(8) $$\mathrm{score}\left(\ell |X\right)=\Delta P\left(\ell |X\right)+\lambda \cdot \Delta S_{\min}\left(\ell |X\right),$$

where ΔP(ℓ∣X)=P(X∪{ℓ})−P(X) and $\Delta S_{\min}\left(\ell |X\right)=S_{\min}\left(X\cup \left\{\ell \right\}\right)-S_{\min}\left(X\right)$. The score in (8) is a compact minimum-based fairness term. Its first term follows the standard coverage-function view, while the second term rewards improvements in the current worst-sensor coverage.

The fairness contribution depends on whether a candidate changes the current minimum coverage across all sensors. If several sensors share the same minimum, improving only one of them leaves $S_{\min}$ unchanged because at least one other sensor still remains at that level. This situation occurs at the start of the ordering, where every sensor has zero coverage and the first selected links may be determined by their aggregate marginal gain. As the ordering develops and fewer sensors remain at the minimum, a link that raises the coverage of the last remaining worst-covered sensor also produces a positive $\Delta S_{\min}$. Score ties that remain after both terms are evaluated are resolved by lexicographic link order. During a shared minimum, the selection is driven by ΔP, so differences in per-sensor observed-packet counts and in the number of packets covered by individual links can influence which sensor receives an additional link first.

**Figure 1:**
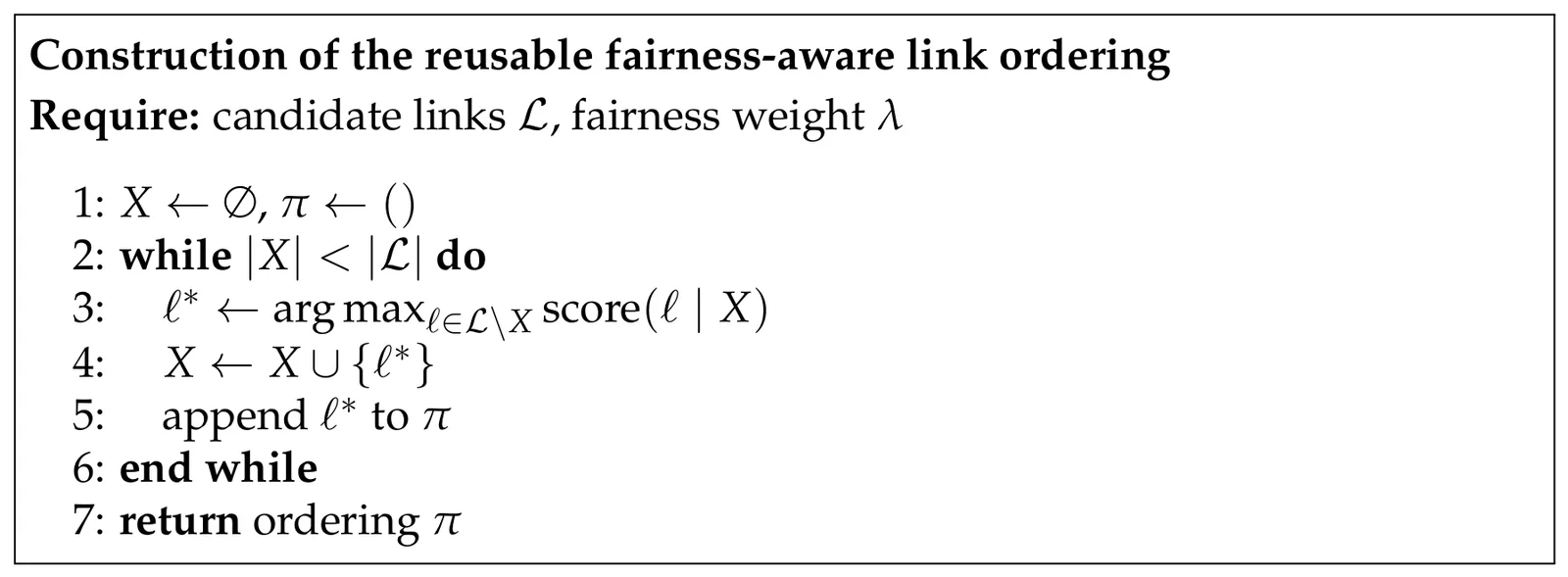
Construction of the reusable fairness-aware link ordering. For a requirement $\left(P_{\min},S_{\min}^{*}\right)$, the returned solution is the shortest prefix of π satisfying both constraints in (5).

A different threshold-aware greedy method, based on per-sensor capped utilities, is described in Section 3.4.3 for comparison. For monotone submodular maximization under a cardinality constraint, the classical greedy algorithm has a (1−1/e) approximation guarantee [21]. This guarantee does not directly apply to (8) because of the additional worst-sensor term, and no approximation ratio is claimed for the fairness-aware ordering used here. The pseudocode is shown in Figure 1.

In the implementation, a complete greedy ordering was constructed once, and the shortest prefix satisfying each threshold pair was reported. Score ties were resolved according to the lexicographic order of link identifiers. Because the score in (8) does not depend on $\left(P_{\min},S_{\min}^{*}\right)$, a single reusable ordering is sufficient for arbitrary service-level agreement (SLA) queries.

#### 3.4.3. Threshold-Aware Multi-Cover Greedy Method

For comparison, a threshold-dependent capped-utility greedy baseline is implemented, building on formulations of minimum multi-submodular cover [17]. Let $T_{P}=\left(P_{\min}/100\right)\cdot \left|P\right|$ and $T_{s}=\left(S_{\min}^{*}/100\right)\cdot \left|P_{s}\right|$ denote the real-valued cap normalizers for the aggregate and per-sensor terms, respectively. Define the capped objective in Equation (9):

(9) $$F\left(X\right)=\min\;\left(\frac{\left|C(X)\right|}{T_{P}},\;1\right)+\sum_{s\in S}\min\;\left(\frac{|C_{s}\left(X\right)|}{T_{s}},\;1\right).$$

Starting from X=Ø, at each step, the link with the largest gain F(X∪{ℓ})−F(X) is added to the set. Gain ties are resolved by the lexicographic order of link identifiers. The procedure stops when $\left|C\left(X\right)\right|\geq N_{P}$ and $|C_{s}\left(X\right)|\geq N_{s}$ for every sensor. The integer targets are used for this stopping test rather than a floating-point equality involving F(X). Since the capped objective depends on $\left(P_{\min},S_{\min}^{*}\right)$, a new ordering is constructed for every requirement, unlike the reusable ordering constructed by the fairness-aware greedy of Section 3.4.2.

#### 3.4.4. Coverage-Only Greedy

As a fairness-oblivious ablation, we also consider the case λ=0 in (8), which reduces the fairness-aware greedy method to iterative selection by the marginal aggregate observed-packet coverage alone. This ordering is threshold-independent as well and is used to isolate the contribution of the fairness term. Unlike static top-*k* rankings, this greedy method is iterative and marginal, so the comparison isolates the effect of explicitly accounting for per-sensor coverage rather than the effect of iterativity alone.

#### 3.4.5. Exact Optimum via Dynamic Programming

The constraint $S_{\min}\left(X\right)\geq S_{\min}^{*}$ is equivalent to an independent lower-bound constraint for every sensor: $S_{s}\left(X\right)\geq S_{\min}^{*}$, s∈S. Since the coverage of sensor *s* depends only on $X\cap L_{s}$, these constraints are sensor-separable. Furthermore, because each packet belongs to exactly one sensor, the sets $C_{s}\left(X\right)$ are disjoint and $\left|C\left(X\right)\right|=\sum_{s\in S}\left|C_{s}\left(X\right)\right|$, so the aggregate coverage constraint also decomposes across sensors.

This gives the problem the structure of a multiple-choice knapsack-type formulation [23]. For each sensor *s*, we enumerate all $2^{|L_{s}|}\leq 8$ subsets of its own links and retain only those satisfying the sensor-level count constraint $|C_{s}\left(Y\right)|\geq N_{s}$. Let $F_{s}$ denote the set of admissible subsets for sensor *s*. For each $Y\in F_{s}$, let |Y| be its cost and let $n_{s}\left(Y\right)=\left|C_{s}\left(Y\right)\right|$ be the number of packets from sensor *s* covered by this subset. Problem (5) is then equivalent to Equation (10):

(10) $$\min_{{\left\{Y_{s}\in F_{s}\right\}}_{s\in S}}\sum_{s\in S}\left|Y_{s}\right|\;s.t.\;\sum_{s\in S}n_{s}\left(Y_{s}\right)\geq N_{P}.$$

This finite formulation is solved exactly by dynamic programming (DP) over the link budget b∈{0,1,…,|L|}: the sensors are processed one by one, and the DP state stores the maximum total number of covered packets achievable with exactly *b* selected links. Within a fixed budget, the implementation retains the state with the largest fitting-window packet count. If this count is also tied, it retains the first reconstruction path under lexicographically sorted sensor and gateway enumeration. This rule makes the reconstructed subset deterministic without implying that the minimum-cardinality solution is unique. Given the enumerated feasible subsets for each sensor, the DP stage has complexity $O(|S|\cdot |L|\cdot 2^{|L_{s}{|}_{\max}})$.

For the three reference requirements, the number of distinct minimum-cardinality solutions was counted exactly by a meet-in-the-middle enumeration over the sensor-level admissible subsets, retaining only combinations whose total cost equals the DP-certified optimal budget and whose aggregate coverage is above the required threshold.

The robust all-month solver uses sensor-local link subsets that satisfy the sensor-level requirement in every month where the corresponding sensor is active. For sensor *s*, let $F_{s}^{R}$ denote this set of admissible subsets, and for $Y\in F_{s}^{R}$, let $n_{s}^{\left(m\right)}\left(Y\right)$ be the number of packets from sensor *s* covered by *Y* in month *m*. The binary variable $z_{sY}$ indicates whether subset *Y* is selected. The optimization used in the implementation is given in Equation (11):

(11) $$\begin{array}{cc} \mathrm{minimise}\; & \sum_{s\in S}\sum_{Y\in F_{s}^{R}}\left|Y\right|z_{sY}\\ \mathrm{subject}\;\mathrm{to}\; & \sum_{Y\in F_{s}^{R}}z_{sY}=1,\;s\in S,\\ \; & \sum_{s\in S}\sum_{Y\in F_{s}^{R}}n_{s}^{\left(m\right)}\left(Y\right)z_{sY}\geq N_{P}^{\left(m\right)},\;m\in M,\\ \; & z_{sY}\in \left\{0,1\right\}. \end{array}$$

The first constraint selects one admissible local subset for every sensor, while the monthly aggregate constraint requires their combined packet coverage to reach $N_{P}^{\left(m\right)}$ in every month. The model is solved with scipy.optimize.milp using a zero relative mixed-integer programming gap, after which the reconstructed link set is checked directly against all monthly aggregate and sensor-level coverage requirements.

### 3.5. Temporal Fitting and Evaluation Protocol

The greedy and exact-DP methods described in Section 3.4.2, Section 3.4.3, Section 3.4.4 and Section 3.4.5 are fitted on different observation windows in the temporal experiments: one calendar month for retrospective reoptimization, and past-only windows for the rolling prospective policies. The robust all-month static benchmark is instead obtained by solving problem (6) once over all 17 months. A selected subset is evaluated either on the same month in the retrospective setting or on the immediately following month in the prospective setting.

The temporal analysis uses the 17 calendar months of the UVA dataset. The set of active sensors is determined separately for each month, as described in Section 3.1, so that sensor10, which first transmits in May 2025, is treated as absent from the active-sensor set in all earlier months. The analysis combines two retrospective or static references with three prospective policies:

- **Retrospective same-month reoptimization.** For each month *m*, the exact DP, the proposed greedy, the multi-cover greedy and the coverage-only greedy are fitted and evaluated on month *m*. The exact result is also used as the same-month oracle in the prospective comparison.
- **All-month robust static optimization.** Problem (6) is solved once over all months and returns a single subset that must remain feasible throughout the observation window.
- **Previous-month policy.** For test month *m*, the subset is fitted only on month m−1.
- **Expanding-window policy.** For test month *m*, the subset is fitted on all months preceding *m*.
- **Frozen-initial-window policy.** A subset is fitted once on February–April 2024 and retained for all subsequent test months.

Test months for the rolling policies are the 16 months following February 2024. The three prospective policies and the same-month oracle are compared on a common 14-month evaluation window from May 2024 through June 2025, beginning when the frozen-initial-window policy first becomes available.

Pass rate, selected-link budget and reconfiguration metrics are reported in the main text. The per-month output additionally records the excess budget over the same-month oracle and the subset Jaccard similarity for reproducibility and supplementary analysis (see Supplementary Tables S1 and S2). The reported metrics are defined as follows:

- **Pass rate**—the fraction of test months in which both thresholds are satisfied.
- **Selected-link budget**—the number of links in the fitted subset.
- **Reconfigurations**—the number of transitions at which the fitted subset differs from the previous subset.
- **Maximum links changed in one reconfiguration**—the largest symmetric difference between two consecutive subsets.

### 3.6. Sensitivity Analyses

To check whether the incomplete boundary months affected the robust static solution, the all-month optimization was repeated after removing February 2024 and June 2025. The remaining window contains 15 complete calendar months, with the candidate links, coverage requirements and optimization procedure kept unchanged from the main analysis, which allows the resulting subset to be compared directly with the original 17-month solution.

The sensitivity of the frozen-initial-window policy to the choice of its initial fitting window was examined separately, motivated by the incomplete first calendar month. The original three-month fitting window, February–April 2024, was replaced by March–May 2024, the first three complete calendar months. To preserve a common prospective evaluation window, both fitted subsets were then evaluated on June 2024–June 2025. The fairness-weight sensitivity was examined separately under a more uneven distribution of observed-packet volumes across sensors. The balanced synthetic cases use 500 observed packets for every sensor, while the heterogeneous cases keep the same total number of packets and distribute them according to the per-sensor observed-packet proportions measured in the UVA data. For synthetic deployments larger than ten sensors, the empirical ten-sensor profile is repeated and randomly permuted across the generated sensors, while paired balanced and heterogeneous cases created from the same seed retain the same sampled link-coverage profiles.

Integer packet counts were obtained using the largest-remainder allocation method. Each synthetic sensor was initially assigned one packet. The remaining packet budget was allocated proportionally to the empirical weights by taking the integer parts of the allocations and assigning any residual packets to sensors with the largest fractional remainders. The experiment uses 10, 50 and 100 sensors with three gateways, 5 random seeds, the 3 reference requirements and λ∈{0,0.01,0.02,0.1,1.0}, with the exact DP used as the cardinality reference for every generated case. The threshold-dependent multi-cover greedy method was additionally evaluated on the same heterogeneous instances for comparison.

### 3.7. Implementation and Reproducibility

All methods were implemented in Python 3.12.3 using NumPy 2.3.5, pandas 2.3.2 and SciPy 1.17.0. The binary integer linear programme of (6) was solved with the HiGHS backend exposed through scipy.optimize.milp. Runtime measurements were taken on an AMD Ryzen 5 5600X 6-Core Processor with 16 GB of RAM running Microsoft Windows 10 Pro, with the reception patterns precomputed in memory. For each requirement, the exact DP was executed 100 times, and the median and 95th-percentile runtimes were recorded. The measurements exclude data loading and reception-pattern construction.

For the studied deployment, the exact DP applied to precomputed reception patterns required a median runtime of 0.33–0.37 ms over 100 runs per requirement. The 95th-percentile runtime ranged from approximately 0.35 to 0.39 ms. The exact DP obtains the solution without invoking an external integer linear programming (ILP) solver.

The analysis code, the derived reception-pattern tables and the scripts that regenerate the tables and figures reported in this article are available in the repository specified in the Data Availability Statement. Generative artificial intelligence was not used to generate or modify the data, numerical results or figures.

## 4. Results

Reference results are first reported for three requirements: $\left(P_{\min},S_{\min}^{*}\right)=\left(90,80\right)$, (95,85), (98,90). The evaluation is then extended to a grid of 1000 threshold pairs. The proposed fairness-aware ordering uses λ=1.0 unless otherwise specified.

### 4.1. Spatial Compression

**Figure 2:**
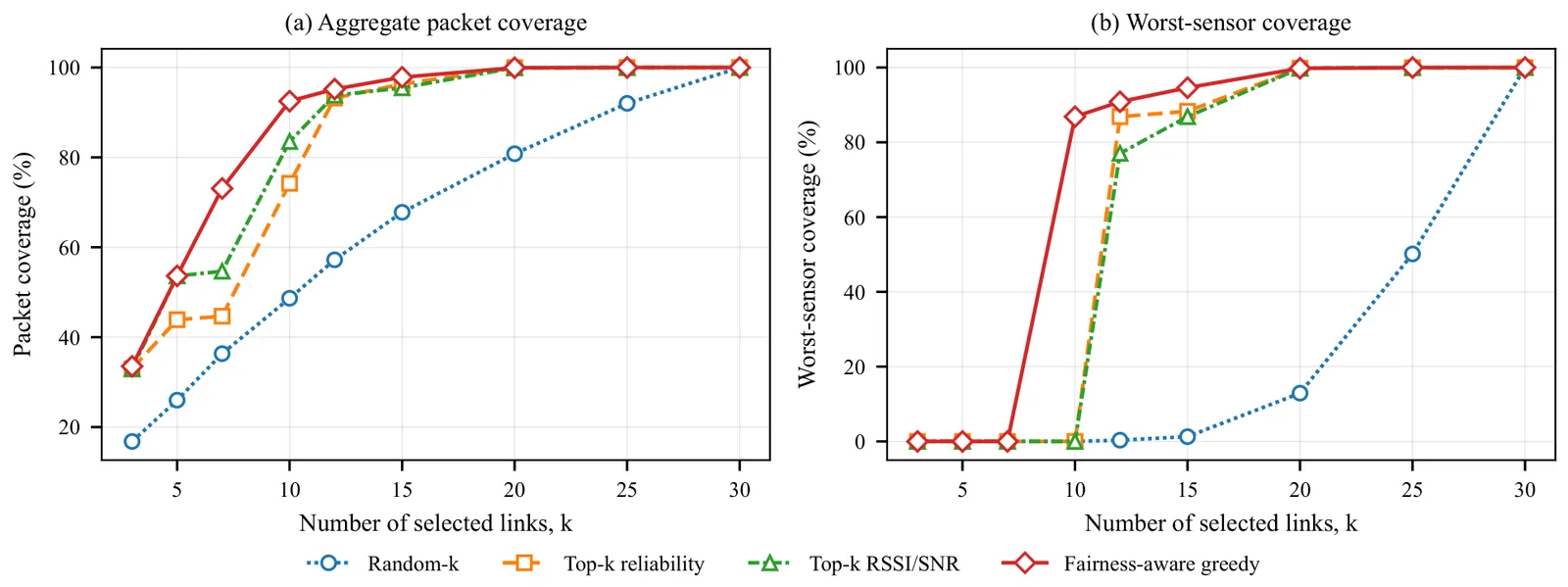
Comparison of the proposed fairness-aware ordering with random and static ranking baselines: (**a**) aggregate observed-packet coverage; (**b**) worst-sensor coverage. At k=10, the proposed ordering reaches 92.5% aggregate and 86.9% worst-sensor coverage, whereas both deterministic top-*k* rankings leave at least one sensor completely uncovered.

Figure 2 shows how aggregate and worst-sensor coverage vary with the link budget. At k=10, the proposed fairness-aware ordering achieves 92.5% aggregate observed-packet coverage and 86.9% worst-sensor coverage. The static top-*k* reliability and RSSI/SNR rankings retain 74.2% and 83.5% of the packets, respectively, but both leave at least one sensor completely uncovered. Thus, a link subset may retain a substantial fraction of all packets while providing zero coverage for an individual sensor.

Table 1 compares the three greedy strategies with the exact DP for the reference requirements. The proposed ordering and the threshold-dependent multi-cover greedy method both match the exact minimum of 10, 12 and 16 links. In contrast, the coverage-only greedy method requires 18 links for all three requirements, corresponding to gaps of 8, 6 and 2 links, respectively. For comparison, the static reliability ranking requires 12, 14 and 18 links, while the RSSI/SNR ranking requires 13, 15 and 19 links.

### 4.2. Wide-Grid Comparison with the Exact Reference

Integer threshold pairs $P_{\min}\in \left\{80,\ldots ,99\right\}$ and $S_{\min}^{*}\in \left\{50,\ldots ,99\right\}$ were evaluated, giving 1000 SLA combinations. All 1000 pairs were feasible for the studied dataset. Both the proposed fairness-aware ordering and the threshold-dependent multi-cover greedy method matched the exact minimum cardinality for all 1000 tested threshold pairs. The coverage-only greedy method matched the exact minimum cardinality for 40 pairs (4%), with a mean gap of 5.84 links and a maximum gap of eight links. On the studied deployment, explicitly accounting for per-sensor coverage distinguished both successful greedy strategies from the aggregate-coverage-only ranking.

The exact optimum was not necessarily unique. For the three reference requirements (90%, 80%), (95%, 85%) and (98%, 90%), the number of distinct exact-optimal subsets was 16, 1 and 13, respectively. Consequently, comparisons across methods are made in terms of exact-optimal cardinality rather than identity with a single reconstructed subset.

### 4.3. Sensitivity to the Fairness Weight

**Figure 3:**
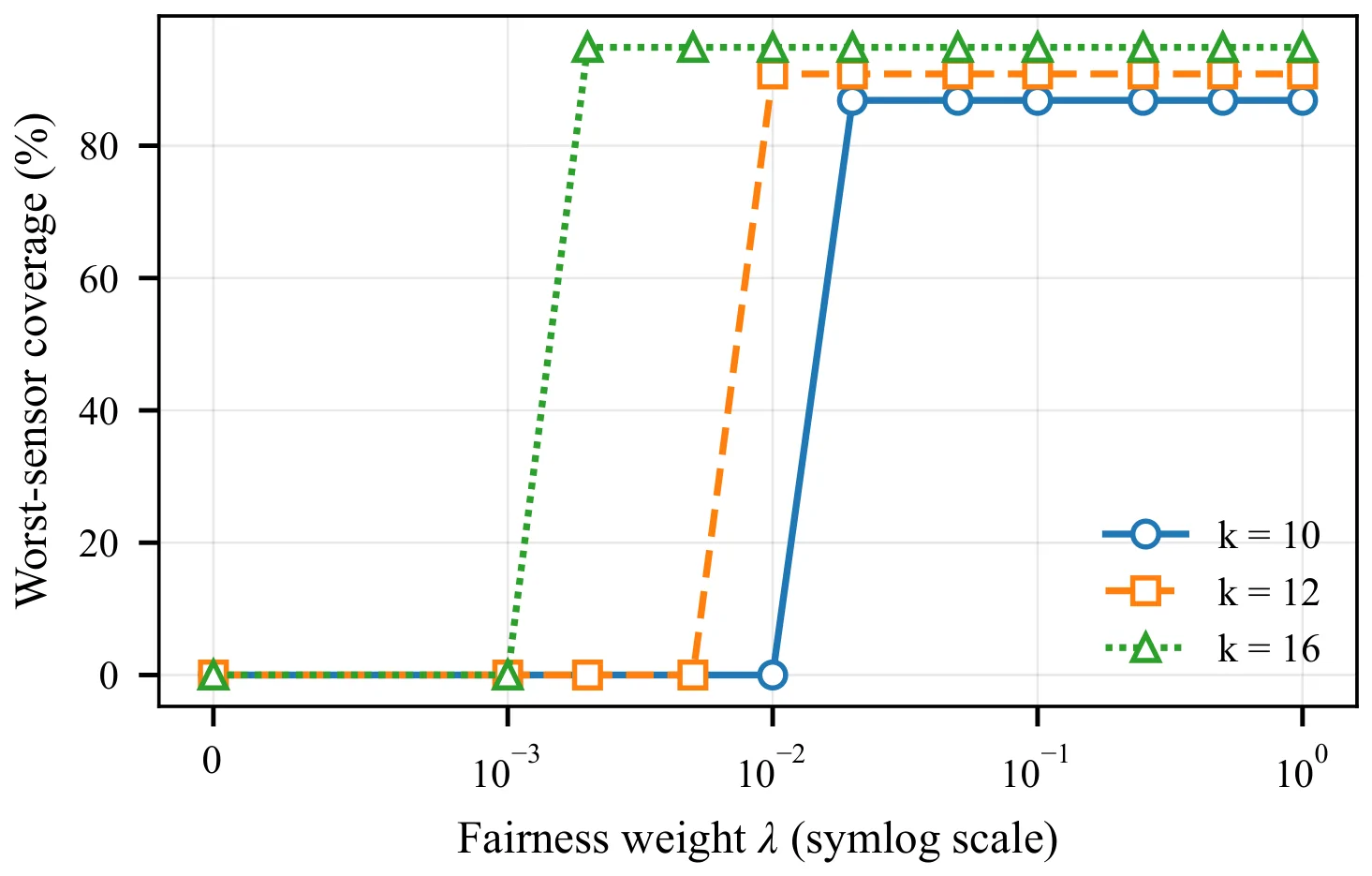
Ablation for the fairness weight λ. Across the three tested budgets, the smallest tested activation value ranges from $2\cdot 10^{-3}$ to $2\cdot 10^{-2}$. Beyond the corresponding value, further increases in λ do not change the selected subset on the tested grid.

An ablation study was conducted on the grid λ∈ {0, $10^{-3}$, $2\cdot 10^{-3}$, $5\cdot 10^{-3}$, $10^{-2}$, $2\cdot 10^{-2}$, $5\cdot 10^{-2}$, $10^{-1}$, 0.25, 0.5, 1.0}. Figure 3 shows the resulting worst-sensor coverage for the three tested budgets.

The results show a sharp transition on the tested λ grid, with worst-sensor coverage rising from 0% to approximately 86.9–94.9% over a relatively narrow range of values. The first tested value associated with this change depends on the link budget and is approximately $2\cdot 10^{-2}$ for k=10, $10^{-2}$ for k=12 and $2\cdot 10^{-3}$ for k=16. On the UVA data, λ=0.02 is already large enough to reach the stable selected subset for all three budgets, and no further subset changes appear over the remainder of the tested grid. A separate experiment with heterogeneous observed-packet volumes was therefore added to examine how this behavior changes when the contribution of individual sensors to the packet set becomes much less uniform.

The ablation further shows that the spread metric $\mathrm{gap}\left(X\right)={\mathrm{median}}_{s\in S}S_{s}\left(X\right)-S_{\min}\left(X\right)$ decreases from approximately 94.1–99.9 percentage points at λ=0 to approximately 4.3–7.2 percentage points once the corresponding tested activation value is reached. This indicates a substantial reduction in the disparity between the median and the worst-sensor coverage.

### 4.4. Fairness-Weight Sensitivity Under Heterogeneous Observed-Packet Volumes

The observed-packet-volume distribution obtained from the UVA data is uneven, partly because sensor10 appears only near the end of the observation period (Table 2). The other 9 sensors contribute approximately 128,000–159,000 observed packets each, whereas only 5514 packets are available for sensor10. When this distribution is transferred to the synthetic cases, the median coefficient of variation of the sensor packet counts is 0.325 and the median ratio between the largest and smallest observed-packet counts is 28.09.

The mean gap from the exact minimum decreases from 5.07 links at λ=0 to 1.47 at λ=0.01, then to 0.40 at λ=0.02, and it reaches 0.07 links for both λ=0.1 and λ=1.0. Across the 15 combinations of seed and reference requirement, the two largest tested values match the exact cardinality in 93.3% of cases.

The larger synthetic deployments behave differently under the same observed-packet-volume profile. With 50 sensors, the mean gap remains 25.40 links once λ reaches 0.02, and no further reduction appears at the larger tested values. For 100 sensors, every positive value in the experiment gives a mean gap of 48.80 links. On the same heterogeneous instances, the threshold-dependent multi-cover greedy method matched the exact minimum cardinality in all 45 combinations of deployment size, random seed and reference requirement. Thus, increasing λ alone does not remove the large gaps of the reusable fairness-aware ordering at 50 and 100 sensors. This comparison shows a limitation of the reusable minimum-based score in this heterogeneous setting, while the threshold-dependent multi-cover greedy method adapts separately to each requirement.

### 4.5. Temporal Quality of Equal-Cardinality Subsets

The wide-grid evaluation established that the proposed fairness-aware ordering and the multi-cover greedy method attain the exact minimum cardinality on all 1000 threshold pairs. Equal cardinality does not imply that the two methods select identical subsets. On the 1000 pairs, the two methods selected the same subset in 494 cases (49.4%) and different subsets of the same cardinality in the remaining 506 cases (50.6%).

For the 506 pairs on which the two subsets differ, a temporal quality comparison was carried out by evaluating each subset month by month over the 17 calendar months and comparing its worst-month aggregate slack $\min_{m\in M}\left[P^{\left(m\right)}\left(X\right)-P_{\min}\right]$ and the number of months in which both thresholds are satisfied. Slack differences whose absolute value does not exceed 0.1 percentage points are treated as ties. The proposed ordering attained a larger worst-month aggregate-coverage slack by more than 0.1 percentage points in 437 of the 506 cases, was within the tie tolerance in 32 cases, and had a smaller slack by more than 0.1 percentage points in 37 cases. The worst-sensor slack was identical for all 506 pairs. In terms of the number of months satisfying both requirements, the proposed subset was better in 53 cases and equal in the remaining 453 cases. It was never worse by this measure.

Equal optimal cardinality does not imply equal temporal quality. Although the two methods satisfy the same whole-period link budget, the reusable proposed ordering more often produces a subset with a larger worst-month aggregate-coverage margin.

### 4.6. Retrospective Monthly Reoptimization

Table 3 reports the retrospective month-specific results.

For the three reference requirements, all evaluated greedy methods matched the exact month-specific cardinality in every one of the 17 months. The mean month-specific budgets were 9.24, 9.29 and 11.59 links. The worst-month budgets were 13, 13 and 15 links. All 17 months were feasible under each requirement.

The retrospective experiment addresses a different question from the whole-period wide-grid evaluation. It considers only three reference requirements and refits every method independently for each month. All four methods attain the same monthly link budget under these conditions, although equal cardinality does not imply identical selected subsets or coverage margins.

### 4.7. Robust All-Month Static Selection

Table 4 brings together the retrospective month-specific results, the whole-period optimum and the robust all-month static optimum defined by (6).

For all three requirements, the mean month-specific budget is smaller than the whole-period optimum. The robust all-month optimum, in contrast, was 19 links out of 30 candidate links for all three requirements.

When February 2024 and June 2025 were removed from the robust optimization, all three reference requirements still required 19 links. The optimization also returned exactly the same selected link set as in the 17-month analysis for each requirement, so the Jaccard similarity between the corresponding solutions was 1.0.

For the three tested requirements, the robust optimum remained at 19 links despite the increasing thresholds. This pattern is specific to the studied threshold set and deployment. The main difference is between the temporal formulations: the mean month-specific optimum uses approximately 9–12 links, whereas a single subset required to remain feasible in every observed month uses 19 links. The latter is approximately twice the retrospective monthly mean for the two lighter requirements and 18.8% larger than the strictest whole-period optimum.

### 4.8. Prospective Rolling Policies

The rolling prospective evaluation covers the 14-month common test window described in Section 3.5. For each test month, a subset is fitted on past reception patterns according to the previous-month, expanding-window or frozen-initial-window policy and is evaluated on the reception patterns of the test month. The same-month oracle provides a retrospective feasibility and minimum-budget reference. It satisfies every test-month requirement by construction, but it is not available to a prospective policy.

Table 5 reports the pass rates, mean link budgets and reconfiguration counts for the proposed ordering.

The three policies differ sharply in reconfiguration behavior and future-month pass rate. The previous-month policy is the most reactive and the least stable: it performs 11 reconfigurations across the 13 transitions for the two lighter requirements and changes the subset at all 13 transitions for the strictest requirement, with a maximum symmetric difference of 7 or 8 links between consecutive subsets. The expanding-window policy uses a smaller mean budget at (90, 80) and larger budgets at the two stricter requirements, but it changes the subset at only 3 or 4 of the 13 transitions, with at most 2 links changed per update. The frozen-initial-window policy does not reconfigure by construction, and the subset fitted on February–April 2024 contains 14 links for the strictest requirement and remains feasible in 10 of the 14 test months, while the 9-link subset obtained for the lightest requirement fails in all 14 months. A sensitivity check replaced the February–April 2024 fitting window with March–May 2024, the first three complete calendar months, and compared both frozen subsets over the common June 2024–June 2025 test period. The selected subsets were unchanged for the (90%, 80%) and (95%, 85%) requirements, with pass rates of 0% and 30.8%, respectively. For the strictest (98%, 90%) requirement, the selected budget decreased from 14 to 13 links and the pass rate decreased from 69.2% to 30.8%, although the two subsets remained highly similar with a Jaccard similarity of 0.929. The comparatively high frozen-policy pass rate at the strictest requirement is therefore sensitive to the particular initial fitting window.

The multi-cover greedy method behaves similarly to the proposed ordering when fitted under the expanding-window policy (pass rates 0.786, 0.429 and 0.500 for the three requirements) and slightly better for the previous-month policy (pass rates of 0.714, 0.500 and 0.286). These differences reflect the subsets selected on the training windows. No single policy dominates across all three requirements and metrics, indicating a trade-off between link-budget efficiency, future-month SLA satisfaction and reconfiguration stability.

Table 6 isolates the effect of the fitting method under the expanding-window policy.

The exact past-window results are obtained from a minimum-cardinality subset fitted on each past window. This reference is distinct from the retrospective same-month oracle and does not optimize next-month feasibility. Another equal-cardinality subset can transfer better to the following month. At (90, 80) and (98, 90), the proposed and coverage-only methods attained the same pass rate, but the proposed ordering used fewer links on average. At (95, 85), the proposed method additionally passed one more test month, with a pass rate of 0.571 compared with 0.500 for coverage-only.

The proposed expanding-window subsets also changed more smoothly: their maximum symmetric difference was two links for all three requirements, while the coverage-only maximum was 10, 9 and 5 links, respectively. Thus, the fairness term did not yield a universal pass-rate improvement, but it reduced the mean link budget and the maximum size of a subset update relative to coverage-only method in this experiment.

### 4.9. New-Sensor Cold Start

May 2025 is a distinguished test month because sensor10 first appears in that month. All rolling prospective policies fit on past months in which sensor10 does not exist and therefore select no link incident to it. When such a subset is evaluated on May 2025, the worst-sensor coverage of the newly appeared sensor is 0%, and both thresholds fail for all three tested requirements. The same-month oracle is feasible on May 2025 for all three requirements because it fits and evaluates on the same month. The retrospective same-month optimum uses 13 links for (90%, 80%) and (95%, 85%) and 15 links for (98%, 90%).

The cold-start month has a disproportionately large impact on the aggregate pass rates in Table 5. Excluding May 2025 from the common evaluation window (13 months) raises the pass rate of the expanding-window policy for the proposed ordering to 0.846, 0.615 and 0.538 for the three reference requirements, respectively. The previous-month policy improves from 0.643, 0.429 and 0.214 to 0.692, 0.462 and 0.231. The frozen-initial-window policy improves from 0.000, 0.286 and 0.714 to 0.000, 0.308 and 0.769. Removing the cold-start month eliminates one failure for every past-only policy and requirement. Its relative effect is largest for the expanding-window policy under the lightest requirement, but the remaining failures show that new-sensor cold start is not the only source of temporal infeasibility.

The retrospective same-month oracle is feasible on May 2025 under all three requirements, so the failures of the past-only policies arise from the information available to those policies. A deployment that requires SLA guarantees for newly activated sensors therefore has to combine the rolling selector with an explicit discovery rule that temporarily retains all links incident to a newly observed sensor, or applies another conservative provisioning rule, until sufficient reception data are available for the next refit. The same-month oracle provides a lower bound on the link budget required once the new sensor is observed, but this solution is not available to a policy restricted to pre-May data.

### 4.10. Reconfiguration Stability

The reconfiguration counts in Table 5 allow the frequency and magnitude of subset changes to be compared directly. In an implementation where the selected links determine link-specific monitoring or processing priorities, a larger symmetric difference would require more such priorities or rules to be updated between consecutive configurations.

The previous-month policy triggers 11, 11 and 13 reconfigurations across the 13 transitions of the common window for the 3 requirements, with maximum symmetric differences of 8, 8 and 7 links, respectively. Its updates therefore range over a substantial fraction of the whole-period optimum on each event. The expanding-window policy triggers only three or four reconfigurations across the same 14 months and never changes more than two links in a single event, and its update trajectory is thus considerably smoother. The frozen-initial-window policy triggers no reconfigurations by construction and provides a stable, if inflexible, reference.

The three policies therefore represent different operational choices: the expanding-window policy limits the frequency and size of updates, the previous-month policy prioritizes reactivity, and the frozen-initial-window policy eliminates reconfiguration.

### 4.11. Synthetic Scalability Evaluation

To examine computational behavior beyond the scale of the UVA deployment, two synthetic scaling experiments were performed. Reception patterns were generated by sampling per-link conditional coverage probabilities, with replacement, from the 30 empirical sensor–gateway coverage fractions observed in the UVA data.

Sampled profiles whose probabilities summed to at most one were rejected. For each accepted profile, independent Bernoulli reception probabilities were calibrated so that conditioning on reception by at least one gateway reproduced the sampled per-link coverage fractions in expectation.

For each synthetic sensor, 500 packet observations were generated and 5 random seeds were evaluated. The sensor-scaling experiment used |S|∈{10,50,100,200,500} with three gateways. The gateway-scaling experiment fixed |S|=50 and used |G|∈{3,5,10,20}. The synthetic data reflect the empirical distribution of per-link coverage in the UVA data, but dependencies among gateway receptions are not modeled.

Table 7 summarizes the scalability results. With three gateways, both methods remained computationally practical as the number of sensors increased, although their runtimes grew with deployment size. The gateway-scaling experiment exposes the exponential cost of local subset enumeration in the exact method. At 20 gateways, the exact method was not executed because sensor-level enumeration would require $2^{20}=1,048,576$ local subsets per sensor, whereas the proposed ordering remained in the millisecond range.

Across all balanced synthetic scalability configurations and seeds for which the exact reference was computed, the proposed ordering differed from the certified optimum by at most one selected link.

## 5. Discussion

### 5.1. Deployment Scale

At fixed small |G| sensor-separability allowed the exact DP to remain tractable up to |S|=500 while increasing the number of gateways made the local enumeration progressively more expensive because each sensor contributes $2^{\left|G\right|}$ possible link subsets.

The behavior of the greedy ordering also depends on the observed-packet-volume distribution used in the synthetic data. When every sensor contributes the same number of packets, the proposed ordering stays within one link of the exact minimum in all configurations where the exact reference is available. After the empirical imbalance in observed-packet volumes is introduced, increasing λ still gives a strong improvement for 10 sensors, but the gaps at 50 and 100 sensors remain large and do not disappear over the tested range. On the same heterogeneous instances, the threshold-dependent multi-cover greedy method matched the exact minimum in all 45 tested cases. This indicates that tuning λ alone does not remove the limitation of the reusable minimum-based ordering in these heterogeneous cases.

The synthetic generator retains marginal link-coverage characteristics from the UVA data, but gateway receptions are generated independently and their full joint structure is not reproduced. These experiments provide controlled comparisons of runtime and selection behavior as network size and the distribution of observed-packet volumes change, while evaluation on larger measured LoRaWAN deployments is still needed.

### 5.2. Alternative Fairness Metrics

This work uses a strict lower bound on the coverage of the worst-performing sensor instead of aggregated metrics such as Jain’s fairness index [24] or other approaches to fair optimization [18,19]. This choice is driven by an engineering interpretation of the SLA, where the loss of coverage for a single sensor is unacceptable even when the total coverage of the network is high. Comparing different fairness metrics for the LoRaWAN link-graph compression problem remains a separate area for future work.

### 5.3. Whole-Period Versus Temporal Formulations

The whole-period compression problem (5) aggregates reception patterns across all months and returns a minimum-cardinality subset satisfying the whole-period aggregate and per-sensor coverage requirements, but this subset is not necessarily robust to month-level variability. The retrospective month-specific results and the robust all-month static results bracket the whole-period optimum from below and above: the mean per-month cardinality is 9.24–11.59 across requirements, the whole-period cardinality is 10–16, and the robust all-month cardinality is 19 for all three requirements. The retrospective month-specific optimum is a lower-bound benchmark, not a directly deployable policy, because it uses the reception patterns of the month on which it is evaluated. The prospective results show that a subset inferred only from past months does not consistently attain this bound. The 19-link all-month solution instead represents the smallest static subset that was verified to remain feasible throughout the full 17-month observation window.

### 5.4. Trade-Offs of Rolling Prospective Policies

The prospective evaluation exposes a trade-off between link-budget efficiency, future-month SLA satisfaction and reconfiguration stability. None of the tested past-only policies dominates on all three requirements, and each corresponds to a distinct operational profile. The previous-month policy prioritizes reactivity at the cost of frequent large-scale reconfigurations. The expanding-window policy provides moderate pass rates with only a few small updates over 14 months. The frozen-initial-window policy trades adaptivity for perfect stability. On the original February–April 2024 fit, it attained the highest pass rate for the strictest tested requirement with a constant 14-link subset, but the sensitivity analysis shows that this advantage is not stable to the choice of the initial fitting window. The results should be read as characterizing this trade-off rather than as recommending one policy.

### 5.5. Operational Implications of New-Sensor Cold Start

Because the past-only policies cannot include links incident to a sensor not observed in any training month, a rolling selector alone cannot handle a sensor that first appears in the test month. A deployment that requires SLA guarantees for newly activated sensors therefore has to combine the rolling selector with a discovery rule that temporarily retains all links incident to a newly observed sensor, or otherwise provisions that sensor explicitly, until sufficient data are available for refitting.

### 5.6. Implications for Dependable Urban Sensing

The comparison with the coverage-only ablation demonstrates that aggregate observed-packet coverage alone can mask poor or zero coverage for individual sensors. At k=10, the coverage-only ordering preserves 93.5% of the observed packets while its worst-sensor coverage remains 0%. The static top-*k* rankings likewise leave at least one sensor completely uncovered. When the methods are continued until both requirements are satisfied, the proposed ordering and the multi-cover greedy method attain the exact constrained minimum for the three reference requirements, whereas the coverage-only ordering requires additional links. Per-sensor coverage should be specified explicitly when the loss of observations from any active sensor is operationally unacceptable. The present results do not, however, establish that the per-sensor constraint has zero cost relative to an aggregate-only optimum or that it should be adopted universally across deployments.

### 5.7. Data and Evaluation Limitations

The prospective comparison contains 14 common test months, so one month changes a reported pass rate by approximately 7.1 percentage points, and the dataset contains only one new-sensor cold-start event. The resulting pass rates describe temporal behavior in this deployment and do not provide a population-level estimate of future-month reliability. The frozen-initial-window policy is fitted on February–April 2024, which includes the incomplete first calendar month. Replacing this interval with the first three complete months left the two lighter requirements unchanged but substantially reduced the prospective pass rate for the strictest requirement. This indicates that the frozen-policy result is sensitive to the particular initial fitting window. Separately, removing both incomplete boundary months from the robust all-month formulation left the 19-link solution unchanged for all three requirements.

Packet deduplication relies on the pair (sensor, counter) because the public metadata do not provide a separate protocol-level packet identifier. No repeated pair was found at different timestamps or in different calendar months in the processed data, but this check cannot rule out every possible counter reuse after a device reset or rollover.

Observed-packet coverage is measured relative to uplink packets observed by at least one gateway in the dataset. Packets that were transmitted but missed by all gateways are unavailable and therefore cannot be included in the denominator. Consequently, the reported metric quantifies the preservation of observed packets rather than the end-to-end packet delivery ratio. This denominator convention is used consistently across the whole-period, month-specific, robust and rolling evaluations.

### 5.8. Operational Scope and Reconfiguration

The proposed method is evaluated offline, with the selected sensor–gateway relationships used by downstream monitoring, diagnostic or rule-based processing after reception data are collected. Gateway reception and forwarding remain unchanged, so the selection does not affect radio transmissions or duplicate packet flow to the network server. Automatic propagation of selected priorities through network-server interfaces was not implemented. Iqbal and Hamamreh [25] describe an Osmocom-based open-source infrastructure in which network components can be configured and managed directly for IoT deployment. Although their implementation is based on second-generation cellular technology, its software-configurable architecture provides a useful example for future implementation of the present approach. A similar principle could allow selected link-set changes to be propagated automatically to network-server monitoring or processing components. At the studied scale, sub-millisecond DP runtime makes repeated offline optimization for different SLA requirements inexpensive once reception patterns are constructed, although the complete preprocessing and rolling-fitting pipeline was not benchmarked end to end.

## 6. Conclusions

This paper formulated LoRaWAN link-graph compression as a minimum-cardinality problem with simultaneous constraints on aggregate and per-sensor observed-packet coverage. On the studied 30-link deployment, the proposed fairness-aware ordering and the threshold-dependent multi-cover greedy method attained the DP-certified minimum cardinality for all 1000 tested integer threshold pairs, whereas the coverage-only greedy method did so for only 4% of them. The top-*k* reliability and top-*k* RSSI/SNR rankings required 2–3 more links than the proposed ordering and the multi-cover greedy method for the three reference requirements. On the UVA data, the first tested value producing nonzero worst-sensor coverage ranged from $2\cdot 10^{-3}$ to $2\cdot 10^{-2}$ across the three link budgets. The heterogeneous experiment showed that increasing λ substantially reduced the cardinality gap at 10 sensors, but large gaps remained when the same observed-packet-volume profile was extended to 50 and 100 sensors. On the same heterogeneous instances, the threshold-dependent multi-cover greedy method matched the exact minimum in all 45 tested cases, showing that tuning λ alone does not remove the limitation of the reusable minimum-based ordering at the larger synthetic scales.

Although the proposed and multi-cover methods had the same optimal cardinality for all 1000 pairs, they selected different subsets for 506 pairs. Using a 0.1-percentage-point tie tolerance, the proposed subsets had a larger worst-month aggregate slack in 437 cases, were within the tolerance in 32 cases and had a smaller slack in 37 cases. They satisfied both requirements in more months in 53 cases and never in fewer months.

The evaluation was then extended to a temporal analysis over 17 calendar months. Under retrospective same-month reoptimization, all three greedy methods matched the exact minimum cardinality on every month, with mean budgets of 9.24, 9.29 and 11.59 links for the three requirements and worst-month budgets of 13, 13 and 15 links. A single link subset that satisfies both thresholds on every month simultaneously required 19 links for all three requirements, which quantifies the cost of temporal robustness without reconfiguration. Under rolling prospective fitting on the 14-month common window, the expanding-window policy for the proposed ordering satisfied both thresholds on 78.6%, 57.1% and 50.0% of test months for the three requirements, with three or four subset reconfigurations of at most two links each. The previous-month policy passed less frequently and reconfigured on the majority of months, and the frozen-initial-window policy exchanged all reactivity for perfect stability. The sensitivity analysis further showed that the apparent advantage of the frozen policy under the strictest requirement depends on the choice of the initial fitting window, whereas the 19-link robust all-month solution remained unchanged after the incomplete boundary months were excluded. The May 2025 cold-start failure cannot be avoided by a selector that has no prior information about the newly activated sensor.

The whole-period and temporal results show that per-sensor observed-packet coverage constraints prevent individual sensors from being lost behind high aggregate coverage, while retrospective month-specific optimization provides a lower bound on the link budget with full knowledge of the evaluated month. The rolling experiments show how future-month SLA satisfaction changes with the allowed reconfiguration. These measured results come from one ten-sensor, three-gateway deployment and remain specific to that network.

The synthetic experiments extend the evaluation to larger networks and heterogeneous observed-packet volumes, revealing large cardinality gaps for the reusable ordering at 50 and 100 sensors. The measured and synthetic evaluations therefore play complementary roles: the UVA dataset characterizes the method on a real multi-gateway deployment, while the synthetic experiments show how its computational behavior and selection quality vary with network size and the per-sensor observed-packet-count distribution. Because the synthetic model preserves marginal coverage from the UVA measurements but not the full dependence between gateway receptions, validation on additional larger measured LoRaWAN deployments is still needed. Future work can examine mandatory-link handling for new sensors and use of the selected link set in network-server monitoring and control. Future work could also investigate alternative threshold-independent link orderings aimed at reducing the cardinality gaps observed at larger synthetic scales under heterogeneous observed-packet volumes while preserving reuse across coverage requirements.

## Figures and Tables

**Table 1:** Minimum number of links required to satisfy each requirement on the whole-period dataset.

Requirement $\left(P_{\min},S_{\min}^{*}\right)$	Coverage-Only (λ=0)	Multi-Cover	Proposed Ordering	Exact Optimum
(90%, 80%)	18	10	10	10
(95%, 85%)	18	12	12	12
(98%, 90%)	18	16	16	16

**Table 2:** Fairness-weight sensitivity under heterogeneous per-sensor observed-packet volumes. Gaps are relative to the exact minimum and aggregated over three requirements and five random seeds.

|S|	λ	Mean Gap	Max Gap	Exact Match Rate
10	0	5.07	9	0.0%
10	0.01	1.47	7	60.0%
10	0.02	0.40	3	86.7%
10	0.10	0.07	1	93.3%
10	1.00	0.07	1	93.3%
50	0	31.20	42	0.0%
50	0.01	27.07	37	0.0%
50	0.02	25.40	37	0.0%
50	0.10	25.40	37	0.0%
50	1.00	25.40	37	0.0%
100	0	63.33	93	0.0%
100	0.01	48.80	72	0.0%
100	0.02	48.80	72	0.0%
100	0.10	48.80	72	0.0%
100	1.00	48.80	72	0.0%

**Table 3:** Retrospective monthly reoptimization over 17 months: mean and worst-month link budget per requirement.

Requirement $\left(P_{\min},S_{\min}^{*}\right)$	Exact Mean	Exact Max	Proposed Mean	Multi-Cover Mean	Coverage-Only Mean
(90%, 80%)	9.24	13	9.24	9.24	9.24
(95%, 85%)	9.29	13	9.29	9.29	9.29
(98%, 90%)	11.59	15	11.59	11.59	11.59

**Table 4:** Whole-period, month-specific and robust all-month link budgets.

Requirement $\left(P_{\min},S_{\min}^{*}\right)$	Whole-Period Exact	Mean Monthly Exact	Worst Monthly Exact	Robust All-Month Static
(90%, 80%)	10	9.24	13	19
(95%, 85%)	12	9.29	13	19
(98%, 90%)	16	11.59	15	19

**Table 5:** Prospective policies for the proposed ordering and the same-month exact oracle on the 14-month common window. Dashes indicate that reconfiguration metrics are not applicable to the retrospective same-month oracle.

Policy	Requirement	Pass Rate	Mean Links	Reconfigurations	Max Changed
Previous-month	(90, 80)	0.643	9.29	11	8
Expanding-window	(90, 80)	0.786	9.07	3	2
Frozen initial	(90, 80)	0.000	9.00	0	0
Same-month oracle	(90, 80)	1.000	9.29	—	—
Previous-month	(95, 85)	0.429	9.36	11	8
Expanding-window	(95, 85)	0.571	10.14	3	2
Frozen initial	(95, 85)	0.286	10.00	0	0
Same-month oracle	(95, 85)	1.000	9.29	—	—
Previous-month	(98, 90)	0.214	11.71	13	7
Expanding-window	(98, 90)	0.500	13.64	4	2
Frozen initial	(98, 90)	0.714	14.00	0	0
Same-month oracle	(98, 90)	1.000	11.50	—	—

**Table 6:** Expanding-window comparison on the 14-month common window. Each method column reports pass rate/mean selected links *k*.

Requirement	Exact Past-Window	Proposed	Multi-Cover	Coverage-Only
$\left(P_{\min},S_{\min}^{*}\right)$	Pass/Mean k	Pass/Mean k	Pass/Mean k	Pass/Mean k
(90%, 80%)	0.786/9.07	0.786/9.07	0.786/9.07	0.786/9.71
(95%, 85%)	0.500/10.14	0.571/10.14	0.429/10.14	0.500/10.64
(98%, 90%)	0.500/13.64	0.500/13.64	0.500/13.64	0.500/13.86

**Table 7:** Synthetic scalability results. Exact-DP runtime is reported as the range of median runtimes across the three reference requirements. The proposed runtime is the median time required to construct the reusable ordering prefix sufficient to satisfy all three reference requirements.

|S|	|G|	Local Subsets per Sensor	Exact-DP Runtime	Proposed Runtime	Max Gap (Links)
*Sensor scaling with |G|=3*					
10	3	8	0.36–0.40 ms	0.69 ms	0
50	3	8	4.47–5.46 ms	6.50 ms	1
100	3	8	16.39–19.75 ms	26.38 ms	1
200	3	8	62.37–78.95 ms	133.15 ms	1
500	3	8	0.42–0.54 s	1.52 s	1
*Gateway scaling with |S|=50*					
50	3	8	4.43–5.43 ms	6.54 ms	1
50	5	32	32.20–36.53 ms	6.75 ms	0
50	10	1024	2.97–3.06 s	6.05 ms	1
50	20	1,048,576	Not run	5.75 ms	—

## Data Availability

The LoRaWAN reception metadata analysed in this study are openly available in the University of Virginia Dataverse at https://doi.org/10.18130/V3/RFTICK, reference number [9]. These data were derived from the following publicly available resource: https://dataverse.lib.virginia.edu (accessed on 13 October 2026). The analysis code, the derived reception-pattern tables and the scripts used to reproduce the reported numerical results and generated figures are openly available at https://github.com/olha-bond/lorawan-link-graph-compression (accessed on 13 October 2026) (DOI: https://doi.org/10.5281/zenodo.23199019).

## Supplementary Materials

The following supporting information can be downloaded at https://www.mdpi.com/article/10.3390/s26206562/s1, Table S1: per-month feasibility and link budget for every policy and requirement; Table S2: excess budget over the same-month oracle and subset Jaccard similarity for every test month.

## Abbreviations

The following abbreviations are used in this manuscript:

DP	Dynamic Programming
ILP	Integer Linear Programming
IoT	Internet of Things
LoRaWAN	Long-Range Wide-Area Network
LPWAN	Low-Power Wide-Area Network
RSSI	Received Signal Strength Indicator
SLA	Service-Level Agreement
SNR	Signal-to-Noise Ratio
UTC	Coordinated Universal Time
UVA	University of Virginia
