# Supplementary material for: Threshold-Aware Link-Graph Compression with Per-Sensor Observed-Packet Coverage Constraints and Temporal Robustness in Urban LoRaWAN Networks
Source: Sensors (Basel). 2026 Oct 16;26(20):6562. doi: 10.3390/s26206562 (PMC13708866; doi:10.3390/s26206562)
Supplement: Supplementary file 1 [file sensors-26-06562-s001.zip › sensors-4605819-supplementary.pdf]

Supplementary Materials

Threshold-Aware Link-Graph Compression with Per-Sensor Observed-Packet Coverage Constraints and Temporal Robustness in Urban LoRaWAN Networks

This supplementary file reports the detailed per-month records underlying the 14-month prospective evaluation window (May 2024–June 2025) in the main manuscript. The reference requirements are $(P_{\min}, S^*_{\min}) = (90\%, 80\%)$, $(P_{\min}, S^*_{\min}) = (95\%, 85\%)$, and $(P_{\min}, S^*_{\min}) = (98\%, 90\%)$. For the three prospective policies, the reported subsets are those produced by the proposed fairness-aware ordering; the same-month oracle is the exact minimum-cardinality solution fitted and evaluated on the corresponding test month.

Notation. k is the number of selected links and $\Delta k = k - k_{\text{oracle}}$. May 2025* denotes the new-sensor cold-start month.

Table S1. Per-month feasibility and selected-link budget for the prospective policies and the same-month exact oracle.

Month	Policy	k	Packet coverage (%)	Worst-sensor coverage (%)	Active sensors	Pass
**Requirement:** $(P_{\min}, S^*_{\min}) = (90\%, 80\%)$						
2024-05	Previous-month	9	94.89	88.04	9	Yes
	Expanding-window	9	91.51	73.35	9	No
	Frozen initial	9	91.51	73.35	9	No
	Same-month oracle	9	96.65	90.37	9	Yes
2024-06	Previous-month	9	95.42	87.10	9	Yes
	Expanding-window	9	90.92	68.67	9	No
	Frozen initial	9	90.92	68.67	9	No
	Same-month oracle	9	96.74	88.48	9	Yes
2024-07	Previous-month	9	95.20	85.66	9	Yes
	Expanding-window	9	94.77	85.66	9	Yes
	Frozen initial	9	90.50	66.60	9	No
	Same-month oracle	9	96.29	88.28	9	Yes
2024-08	Previous-month	9	93.72	78.72	9	No
	Expanding-window	9	95.28	89.03	9	Yes
	Frozen initial	9	91.27	68.67	9	No
	Same-month oracle	9	95.83	89.03	9	Yes
2024-09	Previous-month	9	96.01	86.88	9	Yes
	Expanding-window	9	95.09	86.88	9	Yes
	Frozen initial	9	91.41	71.31	9	No
	Same-month oracle	9	96.44	88.62	9	Yes
2024-10	Previous-month	9	92.85	65.31	9	No
	Expanding-window	9	95.05	88.80	9	Yes
	Frozen initial	9	91.50	72.28	9	No
	Same-month oracle	9	96.95	91.56	9	Yes
2024-11	Previous-month	9	96.93	90.53	9	Yes
	Expanding-window	9	94.50	87.83	9	Yes
	Frozen initial	9	90.68	70.33	9	No
	Same-month oracle	9	96.93	90.53	9	Yes
2024-12	Previous-month	9	93.88	65.05	9	No
	Expanding-window	9	95.76	88.28	9	Yes
	Frozen initial	9	91.89	70.18	9	No
	Same-month oracle	9	97.15	91.64	9	Yes
2025-01	Previous-month	9	94.77	87.04	9	Yes
	Expanding-window	9	93.99	86.62	9	Yes
	Frozen initial	9	90.67	74.21	9	No
	Same-month oracle	9	95.84	89.95	9	Yes
2025-02	Previous-month	9	94.45	79.89	9	No
	Expanding-window	9	93.89	83.11	9	Yes
	Frozen initial	9	90.75	75.78	9	No
	Same-month oracle	9	95.81	89.51	9	Yes

Continued on next page.

Table S1. *Continued*

Month	Policy	k	Packet coverage (%)	Worst-sensor coverage (%)	Active sensors	Pass
2025-03	Previous-month	9	93.32	84.46	9	Yes
	Expanding-window	9	93.18	84.73	9	Yes
	Frozen initial	9	90.42	78.44	9	No
	Same-month oracle	9	96.55	92.74	9	Yes
2025-04	Previous-month	9	95.45	90.67	9	Yes
	Expanding-window	9	93.40	85.13	9	Yes
	Frozen initial	9	88.27	72.69	9	No
	Same-month oracle	9	95.45	90.67	9	Yes
2025-05*	Previous-month	9	73.78	0.00	10	No
	Expanding-window	9	65.13	0.00	10	No
	Frozen initial	9	54.09	0.00	10	No
	Same-month oracle	13	96.98	91.47	10	Yes
2025-06	Previous-month	13	94.58	83.16	9	Yes
	Expanding-window	10	95.45	89.02	9	Yes
	Frozen initial	9	82.29	0.00	9	No
	Same-month oracle	9	95.71	89.02	9	Yes
**Requirement:** $(P_{\min}, S^*_{\min}) = (95\%, 85\%)$						
2024-05	Previous-month	10	96.21	90.37	9	Yes
	Expanding-window	10	94.96	88.04	9	No
	Frozen initial	10	94.96	88.04	9	No
	Same-month oracle	9	96.65	90.37	9	Yes
2024-06	Previous-month	9	95.42	87.10	9	Yes
	Expanding-window	10	94.99	84.52	9	No
	Frozen initial	10	94.99	84.52	9	No
	Same-month oracle	9	96.74	88.48	9	Yes
2024-07	Previous-month	9	95.20	85.66	9	Yes
	Expanding-window	10	94.84	85.66	9	No
	Frozen initial	10	94.84	85.66	9	No
	Same-month oracle	9	96.29	88.28	9	Yes
2024-08	Previous-month	9	93.72	78.72	9	No
	Expanding-window	10	95.35	89.03	9	Yes
	Frozen initial	10	95.35	89.03	9	Yes
	Same-month oracle	9	95.83	89.03	9	Yes
2024-09	Previous-month	9	96.01	86.88	9	Yes
	Expanding-window	10	95.14	86.88	9	Yes
	Frozen initial	10	95.14	86.88	9	Yes
	Same-month oracle	9	96.44	88.62	9	Yes
2024-10	Previous-month	9	92.85	65.31	9	No
	Expanding-window	10	95.11	88.80	9	Yes
	Frozen initial	10	95.11	88.80	9	Yes
	Same-month oracle	9	96.95	91.56	9	Yes
2024-11	Previous-month	9	96.93	90.53	9	Yes
	Expanding-window	10	95.84	87.83	9	Yes
	Frozen initial	10	94.54	87.83	9	No
	Same-month oracle	9	96.93	90.53	9	Yes
2024-12	Previous-month	9	93.88	65.05	9	No
	Expanding-window	10	96.29	88.28	9	Yes
	Frozen initial	10	95.79	88.28	9	Yes
	Same-month oracle	9	97.15	91.64	9	Yes
2025-01	Previous-month	9	94.77	87.04	9	No
	Expanding-window	10	95.28	86.62	9	Yes
	Frozen initial	10	94.03	86.62	9	No
	Same-month oracle	9	95.84	89.95	9	Yes

Continued on next page.

Table S1. *Continued*

Month	Policy	k	Packet coverage (%)	Worst-sensor coverage (%)	Active sensors	Pass
2025-02	Previous-month	9	94.45	79.89	9	No
	Expanding-window	10	94.82	83.11	9	No
	Frozen initial	10	93.93	83.11	9	No
	Same-month oracle	9	95.81	89.51	9	Yes
2025-03	Previous-month	9	93.32	84.46	9	No
	Expanding-window	10	94.70	84.73	9	No
	Frozen initial	10	93.24	84.73	9	No
	Same-month oracle	9	96.55	92.74	9	Yes
2025-04	Previous-month	9	95.45	90.67	9	Yes
	Expanding-window	10	95.05	85.35	9	Yes
	Frozen initial	10	91.83	83.82	9	No
	Same-month oracle	9	95.45	90.67	9	Yes
2025-05*	Previous-month	9	73.78	0.00	10	No
	Expanding-window	10	70.25	0.00	10	No
	Frozen initial	10	61.45	0.00	10	No
	Same-month oracle	13	96.98	91.47	10	Yes
2025-06	Previous-month	13	94.58	83.16	9	No
	Expanding-window	12	97.48	89.69	9	Yes
	Frozen initial	10	84.59	0.00	9	No
	Same-month oracle	9	95.71	89.02	9	Yes
**Requirement:** $(P_{\min}, S^*_{\min}) = (98\%, 90\%)$						
2024-05	Previous-month	14	98.54	95.20	9	Yes
	Expanding-window	14	98.54	95.20	9	Yes
	Frozen initial	14	98.54	95.20	9	Yes
	Same-month oracle	11	98.51	95.20	9	Yes
2024-06	Previous-month	11	97.39	87.10	9	No
	Expanding-window	14	99.02	96.05	9	Yes
	Frozen initial	14	99.02	96.05	9	Yes
	Same-month oracle	11	98.70	96.05	9	Yes
2024-07	Previous-month	11	97.57	91.78	9	No
	Expanding-window	13	98.24	93.46	9	Yes
	Frozen initial	14	98.63	93.46	9	Yes
	Same-month oracle	11	98.46	92.29	9	Yes
2024-08	Previous-month	11	97.64	91.55	9	No
	Expanding-window	13	98.36	95.45	9	Yes
	Frozen initial	14	98.77	95.45	9	Yes
	Same-month oracle	12	98.84	96.94	9	Yes
2024-09	Previous-month	12	99.05	96.86	9	Yes
	Expanding-window	13	98.18	94.02	9	Yes
	Frozen initial	14	98.71	94.02	9	Yes
	Same-month oracle	11	98.54	92.86	9	Yes
2024-10	Previous-month	11	97.79	93.05	9	No
	Expanding-window	13	97.61	91.28	9	No
	Frozen initial	14	98.58	94.57	9	Yes
	Same-month oracle	11	98.26	96.21	9	Yes
2024-11	Previous-month	11	97.84	90.53	9	No
	Expanding-window	13	97.52	87.83	9	No
	Frozen initial	14	98.87	95.22	9	Yes
	Same-month oracle	11	98.56	94.50	9	Yes
2024-12	Previous-month	11	95.38	65.05	9	No
	Expanding-window	13	97.82	88.28	9	No
	Frozen initial	14	99.13	95.67	9	Yes
	Same-month oracle	10	98.03	94.09	9	Yes

Continued on next page.

Table S1. *Continued*

Month	Policy	k	Packet coverage (%)	Worst-sensor coverage (%)	Active sensors	Pass
2025-01	Previous-month	10	95.61	87.04	9	No
	Expanding-window	13	97.22	86.62	9	No
	Frozen initial	14	98.71	92.98	9	Yes
	Same-month oracle	12	98.61	92.98	9	Yes
2025-02	Previous-month	12	98.47	92.07	9	Yes
	Expanding-window	14	98.58	92.07	9	Yes
	Frozen initial	14	98.58	92.07	9	Yes
	Same-month oracle	12	98.73	93.20	9	Yes
2025-03	Previous-month	12	96.90	84.46	9	No
	Expanding-window	14	97.95	88.06	9	No
	Frozen initial	14	97.95	88.06	9	No
	Same-month oracle	11	98.04	94.01	9	Yes
2025-04	Previous-month	11	97.43	94.04	9	No
	Expanding-window	14	97.26	85.35	9	No
	Frozen initial	14	97.26	85.35	9	No
	Same-month oracle	12	98.07	94.76	9	Yes
2025-05*	Previous-month	12	83.75	0.00	10	No
	Expanding-window	14	84.73	0.00	10	No
	Frozen initial	14	79.54	0.00	10	No
	Same-month oracle	15	98.77	93.09	10	Yes
2025-06	Previous-month	15	98.07	86.44	9	No
	Expanding-window	16	99.02	96.51	9	Yes
	Frozen initial	14	86.98	0.00	9	No
	Same-month oracle	11	98.02	92.15	9	Yes

Table S1 note. "Pass" means that both the aggregate packet-coverage threshold and the per-sensor worst-coverage threshold are satisfied on the corresponding test month. The same-month oracle uses the exact method by construction.

Table S2. Excess selected-link budget relative to the same-month exact oracle and subset Jaccard similarity with that oracle for each test month.

Month	Policy	k	Oracle k	Δk	Jaccard
**Requirement:** $(P_{\min}, S^*_{\min}) = (90\%, 80\%)$					
2024-05	Previous-month	9	9	0	0.385
	Expanding-window	9	9	0	0.286
	Frozen initial	9	9	0	0.286
	Same-month oracle	9	9	0	1.000
2024-06	Previous-month	9	9	0	0.636
	Expanding-window	9	9	0	0.500
	Frozen initial	9	9	0	0.500
	Same-month oracle	9	9	0	1.000
2024-07	Previous-month	9	9	0	0.500
	Expanding-window	9	9	0	0.286
	Frozen initial	9	9	0	0.200
	Same-month oracle	9	9	0	1.000
2024-08	Previous-month	9	9	0	0.500
	Expanding-window	9	9	0	0.636
	Frozen initial	9	9	0	0.500
	Same-month oracle	9	9	0	1.000
2024-09	Previous-month	9	9	0	0.636
	Expanding-window	9	9	0	0.385
	Frozen initial	9	9	0	0.286
	Same-month oracle	9	9	0	1.000

Continued on next page.

Table S2. *Continued*

Month	Policy	k	Oracle k	Δk	Jaccard
2024-10	Previous-month	9	9	0	0.500
	Expanding-window	9	9	0	0.500
	Frozen initial	9	9	0	0.385
	Same-month oracle	9	9	0	1.000
2024-11	Previous-month	9	9	0	1.000
	Expanding-window	9	9	0	0.500
	Frozen initial	9	9	0	0.385
	Same-month oracle	9	9	0	1.000
2024-12	Previous-month	9	9	0	0.800
	Expanding-window	9	9	0	0.636
	Frozen initial	9	9	0	0.500
	Same-month oracle	9	9	0	1.000
2025-01	Previous-month	9	9	0	0.636
	Expanding-window	9	9	0	0.636
	Frozen initial	9	9	0	0.500
	Same-month oracle	9	9	0	1.000
2025-02	Previous-month	9	9	0	0.636
	Expanding-window	9	9	0	0.636
	Frozen initial	9	9	0	0.500
	Same-month oracle	9	9	0	1.000
2025-03	Previous-month	9	9	0	0.500
	Expanding-window	9	9	0	0.500
	Frozen initial	9	9	0	0.385
	Same-month oracle	9	9	0	1.000
2025-04	Previous-month	9	9	0	1.000
	Expanding-window	9	9	0	0.636
	Frozen initial	9	9	0	0.385
	Same-month oracle	9	9	0	1.000
2025-05*	Previous-month	9	13	-4	0.467
	Expanding-window	9	13	-4	0.294
	Frozen initial	9	13	-4	0.158
	Same-month oracle	13	13	0	1.000
2025-06	Previous-month	13	9	+4	0.375
	Expanding-window	10	9	+1	0.727
	Frozen initial	9	9	0	0.500
	Same-month oracle	9	9	0	1.000
**Requirement:** $(P_{\min}, S^*_{\min}) = (95\%, 85\%)$					
2024-05	Previous-month	10	9	+1	0.462
	Expanding-window	10	9	+1	0.357
	Frozen initial	10	9	+1	0.357
	Same-month oracle	9	9	0	1.000
2024-06	Previous-month	9	9	0	0.636
	Expanding-window	10	9	+1	0.583
	Frozen initial	10	9	+1	0.583
	Same-month oracle	9	9	0	1.000
2024-07	Previous-month	9	9	0	0.500
	Expanding-window	10	9	+1	0.267
	Frozen initial	10	9	+1	0.267
	Same-month oracle	9	9	0	1.000
2024-08	Previous-month	9	9	0	0.500
	Expanding-window	10	9	+1	0.583
	Frozen initial	10	9	+1	0.583
	Same-month oracle	9	9	0	1.000

Continued on next page.

Table S2. *Continued*

Month	Policy	k	Oracle k	Δk	Jaccard
2024-09	Previous-month	9	9	0	0.636
	Expanding-window	10	9	+1	0.357
	Frozen initial	10	9	+1	0.357
	Same-month oracle	9	9	0	1.000
2024-10	Previous-month	9	9	0	0.500
	Expanding-window	10	9	+1	0.462
	Frozen initial	10	9	+1	0.462
	Same-month oracle	9	9	0	1.000
2024-11	Previous-month	9	9	0	1.000
	Expanding-window	10	9	+1	0.583
	Frozen initial	10	9	+1	0.462
	Same-month oracle	9	9	0	1.000
2024-12	Previous-month	9	9	0	0.800
	Expanding-window	10	9	+1	0.583
	Frozen initial	10	9	+1	0.583
	Same-month oracle	9	9	0	1.000
2025-01	Previous-month	9	9	0	0.636
	Expanding-window	10	9	+1	0.727
	Frozen initial	10	9	+1	0.583
	Same-month oracle	9	9	0	1.000
2025-02	Previous-month	9	9	0	0.636
	Expanding-window	10	9	+1	0.583
	Frozen initial	10	9	+1	0.583
	Same-month oracle	9	9	0	1.000
2025-03	Previous-month	9	9	0	0.500
	Expanding-window	10	9	+1	0.583
	Frozen initial	10	9	+1	0.462
	Same-month oracle	9	9	0	1.000
2025-04	Previous-month	9	9	0	1.000
	Expanding-window	10	9	+1	0.727
	Frozen initial	10	9	+1	0.462
	Same-month oracle	9	9	0	1.000
2025-05*	Previous-month	9	13	-4	0.467
	Expanding-window	10	13	-3	0.353
	Frozen initial	10	13	-3	0.211
	Same-month oracle	13	13	0	1.000
2025-06	Previous-month	13	9	+4	0.375
	Expanding-window	12	9	+3	0.615
	Frozen initial	10	9	+1	0.583
	Same-month oracle	9	9	0	1.000
**Requirement:** $(P_{\min}, S^*_{\min}) = (98\%, 90\%)$					
2024-05	Previous-month	14	11	+3	0.562
	Expanding-window	14	11	+3	0.562
	Frozen initial	14	11	+3	0.562
	Same-month oracle	11	11	0	1.000
2024-06	Previous-month	11	11	0	0.692
	Expanding-window	14	11	+3	0.786
	Frozen initial	14	11	+3	0.786
	Same-month oracle	11	11	0	1.000
2024-07	Previous-month	11	11	0	0.571
	Expanding-window	13	11	+2	0.500
	Frozen initial	14	11	+3	0.562
	Same-month oracle	11	11	0	1.000

Continued on next page.

Table S2. *Continued*

Month	Policy	k	Oracle k	Δk	Jaccard
2024-08	Previous-month	11	12	-1	0.769
	Expanding-window	13	12	+1	0.667
	Frozen initial	14	12	+2	0.733
	Same-month oracle	12	12	0	1.000
2024-09	Previous-month	12	11	+1	0.769
	Expanding-window	13	11	+2	0.500
	Frozen initial	14	11	+3	0.562
	Same-month oracle	11	11	0	1.000
2024-10	Previous-month	11	11	0	0.692
	Expanding-window	13	11	+2	0.500
	Frozen initial	14	11	+3	0.562
	Same-month oracle	11	11	0	1.000
2024-11	Previous-month	11	11	0	0.833
	Expanding-window	13	11	+2	0.600
	Frozen initial	14	11	+3	0.667
	Same-month oracle	11	11	0	1.000
2024-12	Previous-month	11	10	+1	0.750
	Expanding-window	13	10	+3	0.533
	Frozen initial	14	10	+4	0.600
	Same-month oracle	10	10	0	1.000
2025-01	Previous-month	10	12	-2	0.692
	Expanding-window	13	12	+1	0.786
	Frozen initial	14	12	+2	0.857
	Same-month oracle	12	12	0	1.000
2025-02	Previous-month	12	12	0	0.714
	Expanding-window	14	12	+2	0.625
	Frozen initial	14	12	+2	0.625
	Same-month oracle	12	12	0	1.000
2025-03	Previous-month	12	11	+1	0.643
	Expanding-window	14	11	+3	0.667
	Frozen initial	14	11	+3	0.667
	Same-month oracle	11	11	0	1.000
2025-04	Previous-month	11	12	-1	0.917
	Expanding-window	14	12	+2	0.733
	Frozen initial	14	12	+2	0.733
	Same-month oracle	12	12	0	1.000
2025-05*	Previous-month	12	15	-3	0.588
	Expanding-window	14	15	-1	0.611
	Frozen initial	14	15	-1	0.526
	Same-month oracle	15	15	0	1.000
2025-06	Previous-month	15	11	+4	0.625
	Expanding-window	16	11	+5	0.588
	Frozen initial	14	11	+3	0.471
	Same-month oracle	11	11	0	1.000

Table S2 note. $\Delta k = k - k_{\text{oracle}}$. A negative value can occur when a prospective subset uses fewer links than the feasible same-month oracle but fails one or both coverage requirements; it therefore does not indicate a better feasible solution. Jaccard similarity is computed between the prospective subset and the same-month oracle subset. For the oracle itself, $\Delta k = 0$ and Jaccard similarity is 1 by definition.
